# Selective stimulation of catecholamine release from bovine adrenal chromaffin cells by an ionotropic purinergic receptor sensitive to 2-methylthio ATP

**DOI:** 10.1186/1471-2202-8-41

**Published:** 2007-06-20

**Authors:** Ângelo R Tomé, Enrique Castro, Rosa M Santos, Luís M Rosário

**Affiliations:** 1 Center for Neurosciences and Cell Biology, University of Coimbra, Coimbra, Portugal; 2 Department of Biochemistry, Faculty of Sciences and Technology, University of Coimbra, P.O. Box 3126, 3001-401 Coimbra, Portugal; 3 Department of Biochemistry, Molecular Biology and Physiology, Faculty of Medicine and Health Sciences, University of Las Palmas de Gran Canaria, Las Palmas, Spain

## Abstract

**Background:**

2-Methylthioadenosine 5'-triphosphate (2-MeSATP), formerly regarded as a specific P2Y (metabotropic) purinergic receptor agonist, stimulates Ca^2+ ^influx and evokes catecholamine release from adrenal chromaffin cells. These cells express P2Y and P2X (ionotropic) purinoceptors, with the latter providing an important Ca^2+ ^influx pathway. Using single cell calcium imaging techniques, we have determined whether 2-MeSATP might be a specific P2X receptor agonist in bovine chromaffin cells and assessed the relative role of P2X and P2Y receptors on catecholamine secretion from these cells.

**Results:**

ATP raised the [Ca^2+^]_i _in ~50% of the cells. Removing extracellular Ca^2+ ^suppressed the [Ca^2+^]_i_-raising ability of 2-MeSATP, observed in ~40% of the ATP-sensitive cells. This indicates that 2-MeSATP behaves as a specific ionotropic purinoceptor agonist in bovine chromaffin cells. The 2-MeSATP-induced [Ca^2+^]_i_-rises were suppressed by PPADS. UTP raised the [Ca^2+^]_i _in ~40% of the ATP-sensitive cells, indicating that these expressed Ca^2+^-mobilizing P2Y receptors. UTP-sensitive receptors may not be the only P2Y receptors present, as suggested by the observation that ~20% of the ATP-sensitive pool did not respond to either 2-MeSATP or UTP. The average sizes of the ATP- and 2-MeSATP-evoked [Ca^2+^]_i _responses were identical in UTP-insensitive cells. 2-MeSATP stimulated Ca^2+ ^influx and evoked catecholamine release, whereas UTP elicited Ca^2+ ^release from intracellular stores but did not evoke secretion. 2-MeSATP-induced secretion was strongly inhibited by Cd^2+ ^and suppressed by extracellular Ca^2+ ^or Na^+ ^removal. TTX inhibited 2-MeSATP-evoked secretion by ~20%.

**Conclusion:**

2-MeSATP is a specific P2X purinoceptor agonist and a potent secretagogue in bovine chromaffin cells. Activation of 2-MeSATP-sensitive receptors stimulates Ca^2+ ^influx mainly *via *voltage-sensitive Ca^2+ ^channels. For the most part, these are activated by the depolarization brought about by Na^+ ^influx across P2X receptor pores.

## Background

Extracellular ATP plays an important role in catecholamine release from adrenal chromaffin cells, either facilitating cholinergic stimulation via ionotropic (P2X) purinoceptors or inhibiting evoked release through a delayed action on metabotropic (P2Y) purinoceptors (see [1] and references therein). The latter may provide the basis for an auto-inhibitory feedback loop involving both autocrine and paracrine interactions. There are at least seven P2X receptor subunits encoded by distinct genes, which may form homo- or heterotrimeric ionotropic purinoceptors; functional P2X receptors are Ca^2+^-permeable and provide an important Ca^2+ ^influx pathway, both in neurons and other cell types (for review see [2,3]).

The presence of P2X receptor subtypes in chromaffin cells is species-dependent [4] (see [3] for a review). Thus, rat chromaffin cells have either been reported to lack P2X receptors [4] or to show a variable expression of P2X receptor subtypes (P2X_1_, P2X_2_, P2X_5 _and P2X_7_) [5,6]; there is also some evidence that these cells express P2X_4 _receptors in aged animals [7]. Guinea-pig chromaffin cells seem to express P2X_6 _receptors [8], although functional studies point strongly to the presence of P2X_2_-like receptors [4]. There are no studies reporting the expression of specific P2X receptor subtypes in bovine chromaffin cells. However, the presence of P2X receptors in these cells has been suggested by functional studies involving mostly cytosolic free Ca^2+ ^measurements [9-11]. ATP-evoked inward currents have been detected in a limited fraction of the cells [12]. The prevailing P2Y receptor subtype in bovine chromaffin cells seems to be an UTP-sensitive, G_i/o_-coupled P2Y receptor [1,10,13].

ATP and other purinergic agonists evoke catecholamine release from either whole glands or isolated chromaffin cells [9,10,14]. This action is strictly Ca^2+^-dependent, suggesting that it might be mediated by either Ca^2+ ^influx through the receptor-associated pores, opening of voltage-sensitive Ca^2+ ^channels or both. Studying P2X receptor-mediated modulation of chromaffin cell function has been made difficult by the lack of specific agonists and antagonists of P2X receptor subtypes. 2-MeSATP, for example, has been classically regarded as a P2Y receptor agonist; more recently, however, several P2Y receptor subtypes have been found to be insensitive to the ATP derivative [2,3]. Moreover, 2-MeSATP is now known to activate P2X receptor subtypes [15]. There is evidence that 2-MeSATP behaves as a P2X agonist in guinea-pig chromaffin cells, as assessed by its ability to evoke inward currents [4,16]. Previous studies provided conflicting evidence regarding the action of 2-MeSATP on cytosolic free Ca^2+ ^concentration ([Ca^2+^]_i_) in bovine chromaffin cells, with one study reporting sizeable responses [11] and another feeble and sporadic responses [9]. Whether 2-MeSATP might discriminate between P2X and P2Y receptors in chromaffin cells remains unknown.

In this work, we aimed at establishing 2-MeSATP as a specific P2X receptor agonist in bovine chromaffin cells by single-cell calcium imaging. We then investigated the effects of 2-MeSATP and UTP on catecholamine release, aiming at clarifying the relative role of P2X and Ca^2+^-mobilizing P2Y receptors.

## Results

### [Ca^2+^]_i _rises evoked by purinoceptor agonists

[Ca^2+^]_i _changes evoked by ATP receptor agonists were monitored by digital fluorescence imaging of the F_340_/F_380 _fura-2 fluorescence ratio (ΔR). Only cells that displayed sizeable [Ca^2+^]_i _responses to 10 μM 1,1-dimethyl-4-phenylpiperazinium iodide (DMPP), an established agonist of acetylcholine nicotinic receptors in chromaffin cells, were considered for the study. To this end, cells were perifused at the tail of the experiments with 10 μM DMPP for brief periods of time. Our first basic protocol was to stimulate chromaffin cells with ATP, UTP and 2-MeSATP in order to investigate whether UTP-sensitive cells might also be sensitive to 2-MeSATP. Cells were typically subjected to ~60 s pulses of solutions containing each purinoceptor agonist and washed extensively for at least 10 min to minimize receptor desensitization at the time of the next challenge.

**Figure 1:**
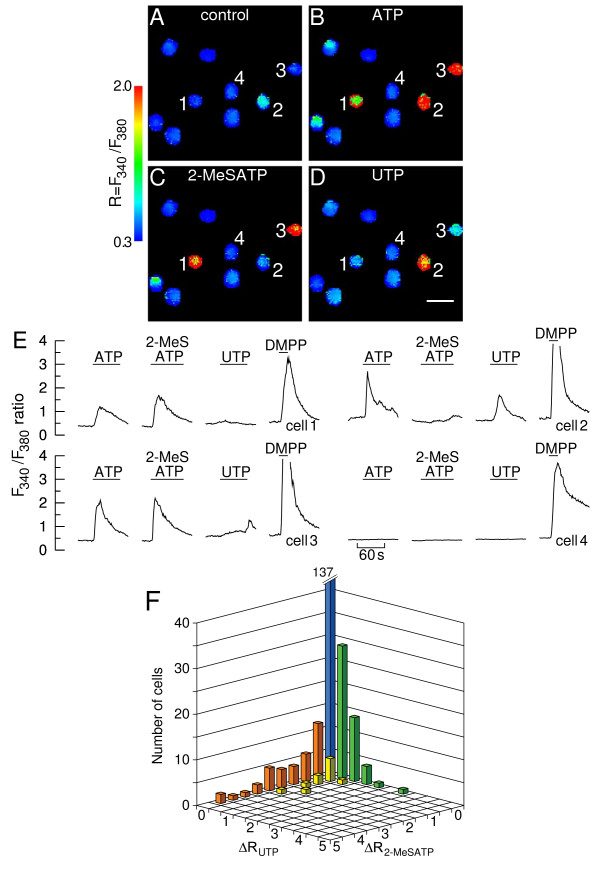
**Calcium responses to ATP, UTP and 2-MeSATP**. **A-D**. Calcium images showing a group of chromaffin cells before (A, "control") and during stimulation with 100 μM ATP (B), 100 μM 2-MeSATP (C) and 100 μM UTP (D). At the end of each experiment cells were stimulated with 10 μM DMPP. Cells were allowed to rest for ~10 min between consecutive stimulations. The fura-2 fluorescence ratio F_340_/F_380 _was determined for each cell in a field on a pixel-by-pixel basis. Images were coded in pseudocolor to show differences in the F_340_/F_380 _ratio. The images corresponding to agonist stimulation (B-D) were captured at the response peaks. Calibration bar: 50 μm; **E**. Time courses of changes in F_340_/F_380 _fluorescence ratio for a 2-MeSATP-sensitive/UTP-insensitive cell (cell 1), an UTP-sensitive/2-MeSATP-insensitive cell (cell 2), a cell displaying a mixed response (cell 3) and an ATP-insensitive cell (cell 4). The lines denote superfusions with DMPP or purinergic agonists. Some of the peak responses to DMPP were truncated for scaling reasons; **F**. Frequency distribution histogram of calcium responses. Changes in ΔR = F_340_/F_380 _were determined from the experiment depicted in A-D and 2 similar experiments (n = 234 cells). The column at the origin represents cells that did not respond to either UTP or 2-MeSATP (ΔR < 0.5, n = 137 cells). This column was truncated for scaling reasons. Columns in orange: cells responding to 2-MeSATP only; columns in green: cells responding to UTP only; columns in yellow: cells exhibiting mixed responses.

Typical pseudocolor fura-2 fluorescence images are depicted in Fig. 1(A–D). Representative time courses of [Ca^2+^]_i _changes for selected chromaffin cells are depicted in E. Resting [Ca^2+^]_i _was low prior to stimulation (A, blue). Challenging cells with 100 μM ATP elicited sizeable, albeit variable peak [Ca^2+^]_i _responses from a large pool of chromaffin cells (B and E; green, yellow or red). It is noteworthy that some cells did not respond to ATP (e.g. cell 4). Several cells (e.g. cell 1) exhibited pronounced [Ca^2+^]_i _rises in response to ATP but not 100 μM UTP. Exposure to 100 μM 2-MeSATP invariably increased the [Ca^2+^]_i _in these cells. In contrast, several cells (e.g. cell 2) responded to both ATP and UTP. For the most part these cells did not respond at all to 2-MeSATP. A fraction of the ATP-sensitive cells responded both to UTP and 2-MeSATP (e.g. cell 3).

The statistical assessment of the [Ca^2+^]_i _responses of chromaffin cells to UTP and 2-MeSATP is presented in Fig. 1F in the form of a 3D histogram. The column at the origin of the histogram (blue) depicts cells that did not respond both to UTP and 2-MeSATP (137 in 234 cells, i.e. 59%); ATP evoked [Ca^2+^]_i _rises in 27 of these cells. Cells displaying a variable response to 2-MeSATP and no UTP response (orange) accounted for approximately 30% of the ATP-sensitive pool; the mean size of the 2-MeSATP-evoked [Ca^2+^]_i _responses was not significantly different from the ATP responses in these cells (1.53 ± 1.22 vs. 1.55 ± 1.18, respectively; n = 37 cells, p = 0.8). UTP-sensitive cells (green) were for the most part (approximately 40% of the ATP-sensitive pool) insensitive to 2-MeSATP, although a minority (ca. 8%, yellow) displayed a mixed sensitivity. The mean sizes of the UTP and ATP responses in UTP-sensitive/2-MeSATP-insensitive cells were 0.71 ± 0.61 and 0.92 ± 0.73, respectively (n = 49 cells; statistically different, p < 0.0001).

**Figure 2:**
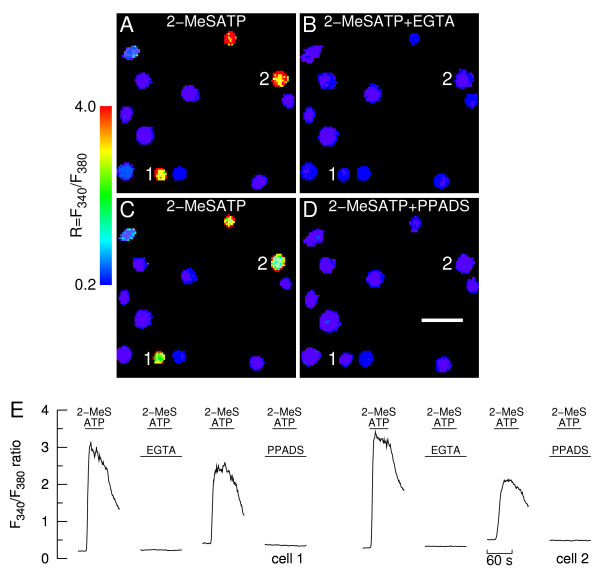
**Effects of PPADS and extracellular Ca^2+ ^removal on calcium responses to 2-MeSATP**. **A-B**. Calcium images showing a group of chromaffin cells during stimulation with 20 μM 2-MeSATP in presence of extracellular calcium (A) and during stimulation in the virtual absence of extracellular calcium ("2-MeSATP +EGTA", B); **C-D**. The cells were subsequently superfused with a Ca^2+^-containing solution and challenged with 2-MeSATP, in the absence (C) or presence of 50 μM PPADS ("2-MeSATP +PPADS", D). Calibration bar: 50 μm. More in the legend to Fig. 1(A-D); **E**. Time courses of changes in F_340_/F_380 _fluorescence ratio for two representative cells (cells 1 and 2, also depicted in A-D). The lines denote superfusions with 2-MeSATP, PPADS or EGTA-containing solution. The responses are representative of data from 3 experiments.

None of the cells responding to 2-MeSATP in presence of Ca^2+ ^responded in the virtual absence of extracellular Ca^2+ ^(Fig. 2A,B,E), suggesting that the ATP derivative is a specific P2X receptor agonist in bovine chromaffin cells. We have previously reported that suramin blocked the ATP-evoked [Ca^2+^]_i _responses in cells lacking ATP responses in the virtual absence of extracellular Ca^2+^; suramin did not affect the UTP responses, suggesting that it behaves as a specific P2X receptor antagonist [10]. We have now extended this conclusion to pyridoxalphosphate-6-azophenyl- 2',4'-disulphonic acid (PPADS, 50 μM). Indeed, the drug blocked the 2-MeSATP (20 μM)-evoked [Ca^2+^]_i _rises in all cells tested (n = 9 cells; Fig. 2C,D,E). Moreover, in parallel experiments, PPADS did not affect the UTP-evoked [Ca^2+^]_i _rises in cells displaying no response to 2-MeSATP (0.69 ± 0.33 vs. 0.73 ± 0.32 in absence of PPADS; n = 11 cells, p = 0.1).

### Effects of purinergic agonists on catecholamine secretion

**Figure 3:**
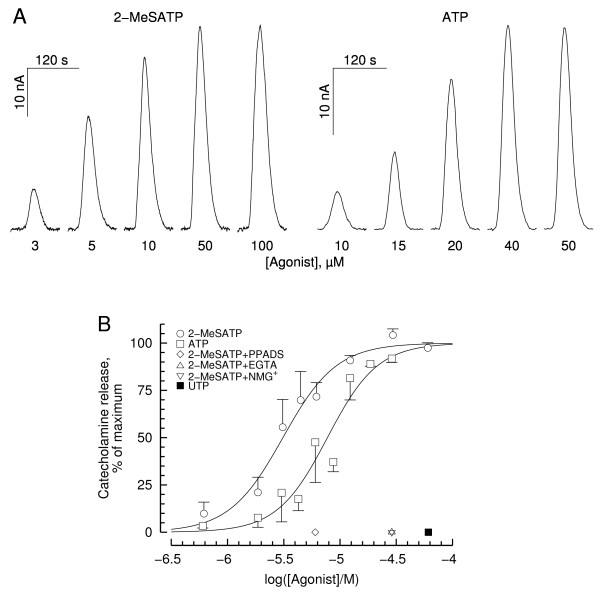
**Effects of 2-MeSATP and ATP on catecholamine release**. **A**. Amperometric currents recorded from chromaffin cell batches in response to brief pulses of increasing 2-MeSATP and ATP concentrations. Cells were allowed to rest for ~10 min between successive applications. Nominal agonist concentrations (in μM) are indicated beneath each trace. Nominal concentrations of test agents are used throughout this legend. Actual concentrations at the cell bed differ from nominal concentrations by a factor of 1.64 (see Methods). Concentrations along the X axis in Fig. 3B are actual concentrations at the cell bed; **B**. Dose-response curves of catecholamine release evoked by 2-MeSATP and ATP (circles and squares, respectively). Data from the experiment depicted in A and two similar experiments. Also depicted are data for UTP (100 μM; filled square) as well as for 2-MeSATP stimulations in presence of 50 μM PPADS (diamond), in the virtual absence of extracellular Ca^2+ ^(EGTA-containing solution, inverted triangle) and in absence of extracellular Na^+ ^(Na^+ ^replacement for NMG^+^, triangle) (n = 3 experiments for each condition).

Both ATP and 2-MeSATP evoked catecholamine secretion in a dose-dependent way, as assessed amperometrically from cell batches (Fig. 3A). In these experiments cells were challenged with brief pulses of either agonist at increasing concentrations, being allowed to rest for ~10 min between successive applications. The concentration-response curves provided in Fig. 3B suggest that 2-MeSATP might be a more potent agonist for purinergic receptors (EC_50 _= 3.1 μM vs. 7.7 μM for ATP; but see the Discussion section for a critical assessment of this hypothesis). (Concentrations of test agents are henceforth given as nominal concentrations. Actual concentrations at the cell bed differ from nominal concentrations by a factor of 1.64, see Methods and legend to Fig. 3.) UTP (100 μM) did not evoke catecholamine secretion. PPADS (50 μM) abolished 2-MeSATP (10 μM)-evoked secretion (Fig. 3B).

Challenging the cells with 50 μM 2-MeSATP in the virtual absence of extracellular calcium failed to elicit secretion (Fig. 3B), indicating that the stimulatory action of the purinoceptor agonist is strictly calcium-dependent. This action may be mediated mostly by activation of voltage-sensitive Ca^2+ ^channels, since 0.5 mM Cd^2+ ^(a blocker of these channels) reduced 2-MeSATP-evoked secretion by 73.0 ± 1.5% (n = 3 experiments); Cd^2+ ^inhibited high K^+ ^(50 mM)-evoked catecholamine secretion by ~98% (n = 3 experiments). Replacing extracellular Na^+ ^for N-methyl-D-glucamine (NMG^+^) abolished 2-MeSATP-evoked secretion (Fig. 3B); tetrodotoxin (TTX, 1 μM) had a modest (18.5 ± 3.0%, n = 3 experiments) inhibitory effect on evoked secretion, suggesting that Na^+ ^influx through TTX-sensitive, voltage-dependent Na^+ ^channels played a minimal role.

## Discussion

We found that ~50% of the cells examined by fluorescence imaging exhibited [Ca^2+^]_i _responses to ATP and that, among these, ~40% yielded positive responses to 2-MeSATP. None of the 2-MeSATP-sensitive cells responded in the virtual absence of extracellular Ca^2+^. Since cell exposure to EGTA-containing solutions caused minimal depletion of intracellular Ca^2+ ^stores [1,10], this implies that 2-MeSATP elicits [Ca^2+^]_i _rises by stimulating Ca^2+ ^influx. Hence, 2-MeSATP behaves as a specific P2X receptor agonist in bovine chromaffin cells.

The 2-MeSATP-sensitive P2X receptors in these cells are blocked by low concentrations of suramin [10] and PPADS (this work). In agreement with a previous study [9], we show here that 2-MeSATP is a stronger secretagogue than ATP. This might suggest that ATP is a weaker agonist of the P2X receptor, in apparent disagreement with the information provided by our former [Ca^2+^]_i _study [11] where the order of potency for purinergic agonists was ATP > 2-MeSATP >> αβ-MeATP, ADP [βS], AMP. We note, however, that activation of a P2Y receptor coupled to G_i/o _inhibits exocytotic release of catecholamines from rat chromaffin cells downstream voltage-sensitive Ca^2+ ^channels and cytosolic Ca^2+ ^elevation [17]. Moreover, activation of P2Y receptors inhibits voltage-sensitive Ca^2+ ^channels via G_i_/G_o _proteins and, thus, depresses Ca^2+^-dependent exocytosis [12,13,18-22]. Subclassification of P2X receptors in bovine chromaffin cells was clearly outside the objectives of the present functional study. Nonetheless, taking into account the sensitivity to inhibitors, as well as the above order of potency for purinergic agonists, P2X receptors expressed in bovine chromaffin cells appear to have the pharmacological profile of P2X_2 _or P2X_5_receptors [2,4,15]. As a note of caution we emphasize that P2X receptors *in situ *most probably are composed of different unit subtypes with a pharmacology distinct from that of the homomeric receptors cloned and transfected to cells, which difficults any attempt at identifying P2X receptor subtypes on the basis of functional studies.

We have also found that ~50% of the ATP-sensitive cells yielded positive responses to UTP. Thus, since UTP is a specific agonist for P2Y receptors in bovine chromaffin cells [1], approximately half of the ATP-sensitive cell population expressed functional Ca^2+^-mobilizing P2Y receptors. This agrees with the finding by Ennion *et al. *that these cells express an as yet unidentified UTP-sensitive, G_i/o_-coupled P2Y receptor [13]. It is noteworthy that the authors also provided evidence for the presence of a G_i/o_-linked, adenine nucleotide-specific P2Y_12 _receptor and detected transcripts for P2Y_1 _receptors by RT-PCR analysis. Accordingly, our results suggest that bovine chromaffin cells express a Ca^2+^-mobilizing, UTP-insensitive P2Y receptor. Indeed, ~20% of the ATP-sensitive pool did not respond to either 2-MeSATP or UTP. Taking into account that P2X receptors are fully sensitive to 2-MeSATP in bovine chromaffin cells, the most likely explanation for this finding is that, indeed, some cells express a Ca^2+^-mobilizing, UTP-insensitive P2Y receptor.

Chromaffin cells exist in the form of two major phenotypes, epinephrine-secreting (adrenergic) and norepinephrine-secreting (noradrenergic) cells [23]. Our previous work [1] showed that P2X receptors and UTP-sensitive P2Y receptors are asymmetrically distributed among two distinct cell pools (noradrenergic and adrenergic cells). The present data are fully compatible with this finding. Indeed, approximately 30% of the ATP-sensitive cells yielded positive responses to 2-MeSATP but not to UTP, suggesting that they express P2X receptors preferentially. Moreover, approximately 40% of the ATP-sensitive cells yielded positive responses to UTP but not to 2-MeSATP, suggesting that they express Ca^2+^-mobilizing P2Y receptors only. A small fraction of the ATP-sensitive cells examined in this study responded both to UTP and 2-MeSATP, suggesting that they co-express P2X and P2Y receptors (see also [10]).

There is evidence that stimulated Ca^2+ ^influx is strongly coupled to catecholamine release; in contrast, Ca^2+ ^release from intracellular stores appears to be loosely coupled to secretion [10,24]. The present data support this concept, inasmuch as P2X receptor activation by 2-MeSATP stimulated Ca^2+ ^influx and evoked catecholamine release, whereas activation of P2Y receptors by UTP elicited Ca^2+ ^release from intracellular stores but did not evoke secretion. We found that, while 2-MeSATP-induced secretion was strictly Na^+^-dependent, the voltage-sensitive Na^+ ^channel blocker TTX [25,26] inhibited evoked release by a modest 20%. This suggests that voltage-sensitive Ca^2+ ^channels were mostly activated by the depolarization brought about by Na^+ ^influx across P2X receptor pores. That voltage-sensitive Ca^2+ ^channels are involved in 2-MeSATP-induced secretion is suggested by the strong inhibitory effect of Cd^2+^. Indeed, Cd^2+ ^blocks Ca^2+ ^influx across these channels without affecting P2X receptors [27,28].

## Conclusion

2-MeSATP is a specific P2X purinoceptor agonist and a potent secretagogue in bovine chromaffin cells. Activation of P2X receptors stimulates Ca^2+ ^influx mainly *via *voltage-sensitive Ca^2+ ^channels. For the most part, these are activated by the depolarization brought about by Na^+ ^influx across P2X receptor pores.

## Methods

### Cell culture

Bovine adrenal glands were obtained from the local slaughterhouse and kept on ice during transportation. Adrenal medulla cells were isolated by collagenase digestion of the glands and purified on a Percoll density gradient essentially as described previously [29,30]. The purified cell fraction thus obtained is enriched in chromaffin cells. Cells were cultured according to established procedures [1]. For the fluorescence imaging experiments, the cells were plated on round (16 mm diameter) glass coverslips coated with poly-L-lysine. For the secretion experiments, the cells were cultured in 60 mm diameter plastic Petri-dishes. Cells were typically used between days 2 and 5 after plating.

### Solutions

The Ca^2+^-containing salt solution used in the imaging and secretion experiments had the following composition (mM): 140 NaCl, 5 KCl, 2 CaCl_2_, 1 MgCl_2_, 10 mM HEPES and 10 glucose (pH 7.4). In some experiments extracellular free [Ca^2+^] was buffered at 100 nM by mixing appropriate amounts of Ca^2+ ^and EGTA, as described elsewhere [31].

### [Ca^2+^]_i _imaging

The coverslips containing the cells were washed in physiological saline supplemented with 1% bovine serum albumin (BSA). The cells were then loaded with 2.5 μM fura-2/AM (the acetoxymethyl ester of fura-2 [32]) for 45 min at 37°C in this medium, under a 95% O_2_/5% CO_2 _atmosphere. Cell handling after loading was carried out following established procedures. Specifically, cells were continuously perifused (approximately 1.5 ml/min) with physiological saline at room temperature. Solution exchange was provided by a four-way stopcock valve located near the recording chamber [1,10,11]. Fluorescence changes were recorded using a multiple excitation MagiCal imaging system (Applied Imaging, U.K.), essentially as described [1,33].

### Catecholamine secretion

Catecholamine secretion from chromaffin cells was measured on-line using a perifusion system similar to that described previously [34]. Approximately 10^6 ^cells were placed in a 0.45 μm flow filter and perifused with the HEPES-containing solution with the aid of a peristaltic pump (Gilson Miniplus 3) at a rate of 1 ml/min. Test drugs were added as brief pulses through a 500-μl loop injector. Taking into account the dead volume of the filter chamber and the flow rate, the actual concentration of a given drug inside the chamber (cell bed) was reduced by a factor of 1.64. Thus, while drug concentrations are given as nominal concentrations throughout the main text and legend to Fig. 3, the concentrations along the X axis in Fig. 3B are corrected for the dilution factor (i.e. they refer to the actual concentrations acting on cells). The effluent solution exiting the filter was driven into an electrochemical detector (set at +500 mV, Omni 90 Potentiostat, Cypress Systems, Lawrence, KS) for direct measurement of catecholamine oxidation current, which was monitored on a chart recorder.

### Data analysis

Data are presented as mean ± S.D. Statistical significance of differences was assessed by paired (within the same experiment) or unpaired (between experiments) Student's t-test; differences were considered significant at the 95% confidence level (P < 0.05).

### Other materials

Fura-2/AM was from Molecular Probes (Eugene, Ore., USA). ATP was from Boehringer (Mannheim, Germany). Unless otherwise specified, all other chemicals were from Sigma Chemical Co. (St. Louis, Mo., USA).

## Authors' contributions

ART carried out the experiments, performed the statistical analysis, prepared the figures, participated in conceiving and designing the study, and helped to draft the manuscript; EC helped in carrying out the experiments and participated in conceiving and designing the study; RMS helped to draft the manuscript; LMR participated in conceiving and coordinating the study and drafted the manuscript. All authors read and approved the final manuscript.
